# Higher Serum Lysophosphatidic Acids Predict Left Ventricular Reverse Remodeling in Pediatric Dilated Cardiomyopathy

**DOI:** 10.3389/fped.2021.710720

**Published:** 2021-08-16

**Authors:** Haichu Wen, Hongzhao You, Yulin Li, Ke Ma, Meng Jiao, Shaowei Wu, Shijie You, Jie Huang, Junwu Su, Yan Gu, Zhiyuan Wang, Ping Zheng, Guanghou Shui, Yuan Wang, Mei Jin, Jie Du

**Affiliations:** ^1^Beijing Institute of Heart, Lung, and Blood Vessel Diseases, Beijing Anzhen Hospital, Capital Medical University, Beijing, China; ^2^State Key Laboratory of Cardiovascular Disease, Cardiovascular Institute, Fuwai Hospital and National Centre for Cardiovascular Diseases, Chinese Academy of Medical Sciences and Peking Union Medical College, Beijing, China; ^3^Department of Pediatric Heart Centre, Beijing Anzhen Hospital, Capital Medical University, Beijing Pediatric Heart Centre, Beijing, China; ^4^Department of Occupational and Environmental Health, School of Public Health, Xi'an Jiaotong University Health Science Center, Xi'an, China; ^5^Department of Clinical Laboratory, Shandong Cancer Hospital and Institute, Shandong First Medical University and Shandong Academy of Medical Sciences, Jinan, China; ^6^State Key Laboratory of Molecular Developmental Biology, Institute of Genetics and Developmental Biology, Chinese Academy of Sciences, Beijing, China

**Keywords:** pediatric dilated cardiomyopathy, left ventricular remodeling reverse, lipid metabolism, lysophosphatidic acids, machine learning

## Abstract

**Background:** The prognosis of pediatric dilated cardiomyopathy (PDCM) is highly variable, ranging from death to cardiac function recovery. Left ventricular reverse remodeling (LVRR) represents a favorable prognosis in PDCM. Disturbance of lipid metabolism is associated with the change of cardiac function, but no studies have examined lipidomics data and LVRR.

**Methods:** Discovery analyses were based on 540 targeted lipids in an observational, prospective China—AOCC (An Integrative-Omics Study of Cardiomyopathy Patients for Diagnosis and Prognosis in China) study. The OPLS-DA and random forest (RF) analysis were used to screen the candidate lipids. Associations of the candidate lipids were examined in Cox proportional hazards regression models. Furthermore, we developed a risk score comprising the significant lipids, with each attributed a score of 1 when the concentration was above the median. All significant findings were replicated in a validation set of the China-AOCC study.

**Results:** There were 59 patients in the discovery set and 24 patients in the validation set. LVRR was observed in 27 patients (32.5%). After adjusting for age, left ventricular ejection fraction (LVEF), and left ventricular end-diastolic dimension (LVEDD) z-score, lysophosphatidic acids (LysoPA) 16:0, LysoPA 18:2, LysoPA 18:1, and LysoPA 18:0 were significantly associated with LVRR in the discovery set, and hazard ratios (HRs) were 2.793 (95% CI, 1.545–5.048), 2.812 (95% CI, 1.542–5.128), 2.831 (95% CI, 1.555–5.154), and 2.782 (95% CI, 1.548-5.002), respectively. We developed a LysoPA score comprising the four LysoPA. When the LysoPA score reached 4, LVRR was more likely to be observed in both sets. The AUC increased with the addition of the LysoPA score to the LVEDD z-score (from 0.693 to 0.875 in the discovery set, from 0.708 to 0.854 in the validation set) for prediction of LVRR.

**Conclusions:** Serum LysoPA can predict LVRR in PDCM patients. When the LysoPA score was combined with the LVEDD z-score, it may help in ascertaining the prognosis and monitoring effects of anti-heart failure pharmacotherapy.

## Introduction

Dilated cardiomyopathy (DCM) is the most common form of pediatric cardiomyopathy and is defined by the presence of left ventricular dilatation and contractile dysfunction. The annual incidence of pediatric dilated cardiomyopathy (PDCM) is 0.57 to 1.13 per 100,000 in children <18 years old (1). Of children, 75–80% present with signs and symptoms of heart failure (2); however, the outcome of PDCM is by no means certain, with 31% death or heart transplantation and 25% of patients regaining normal cardiac function within 1 year, which is defined as left ventricular reverse remodeling (LVRR) (3). Risk factors for death and heart transplantation in PDCM are well studied (4, 5); however, predictors of LVRR are largely unknown.

Left ventricular reverse remodeling is a quantitative and continuous marker assessed by echocardiography, which reflects cardiac function improvement and underlying myocardial viability (6). Additionally, prediction of LVRR indicates other essential clinical implications, especially for monitoring the effects of anti-heart failure pharmacotherapy. Several studies have reported that changes in NT-proBNP level can independently predict LVRR in adults with newly diagnosed DCM and heart failure (7). However, changes in NT-proBNP levels were only associated with adverse events in children with heart failure related to DCM (8). In addition, a small-scale study (9) identified some clinical predictors of LVRR in PDCM, such as higher ejection fraction, younger age, and smaller left ventricular end diastolic z-score. While the use of parameters from echocardiography would be a simple approach, they only reflect a limited aspect of cardiac physiology, and reproducibility of the measurement is relatively poor. Hence, there is still a lack of specific biomarkers to predict LVRR based on pathologic mechanisms of PDCM.

Both heredity and environment factors will affect the pathological process of DCM, eventually reflected as abnormal metabolism (10). Developments in lipidomics, a subfield of metabolomics, provide new insights into biomarkers and disease pathogenesis in cardiovascular diseases (11). The abnormal expression of lipid metabolites not only participates in the energy metabolism of cardiomyocytes (12), but also regulates many cardiac pathological processes, such as myocardial fibrosis (13), cardiomyocyte hypertrophy (14), and apoptosis (15), which provides the molecular basis for lipidomics research in LVRR.

Evidence on the link between lipid profiling in PDCM and LVRR is lacking. Here we used an absolute quantitative metabolomics platform to screen specific serum markers of LVRR and differentiate patients likely to recover by developing a risk score that combines lipid metabolites with left ventricular end-diastolic dimension (LVEDD) z-score.

## Materials and Methods

### Study Population

**Figure 1:**
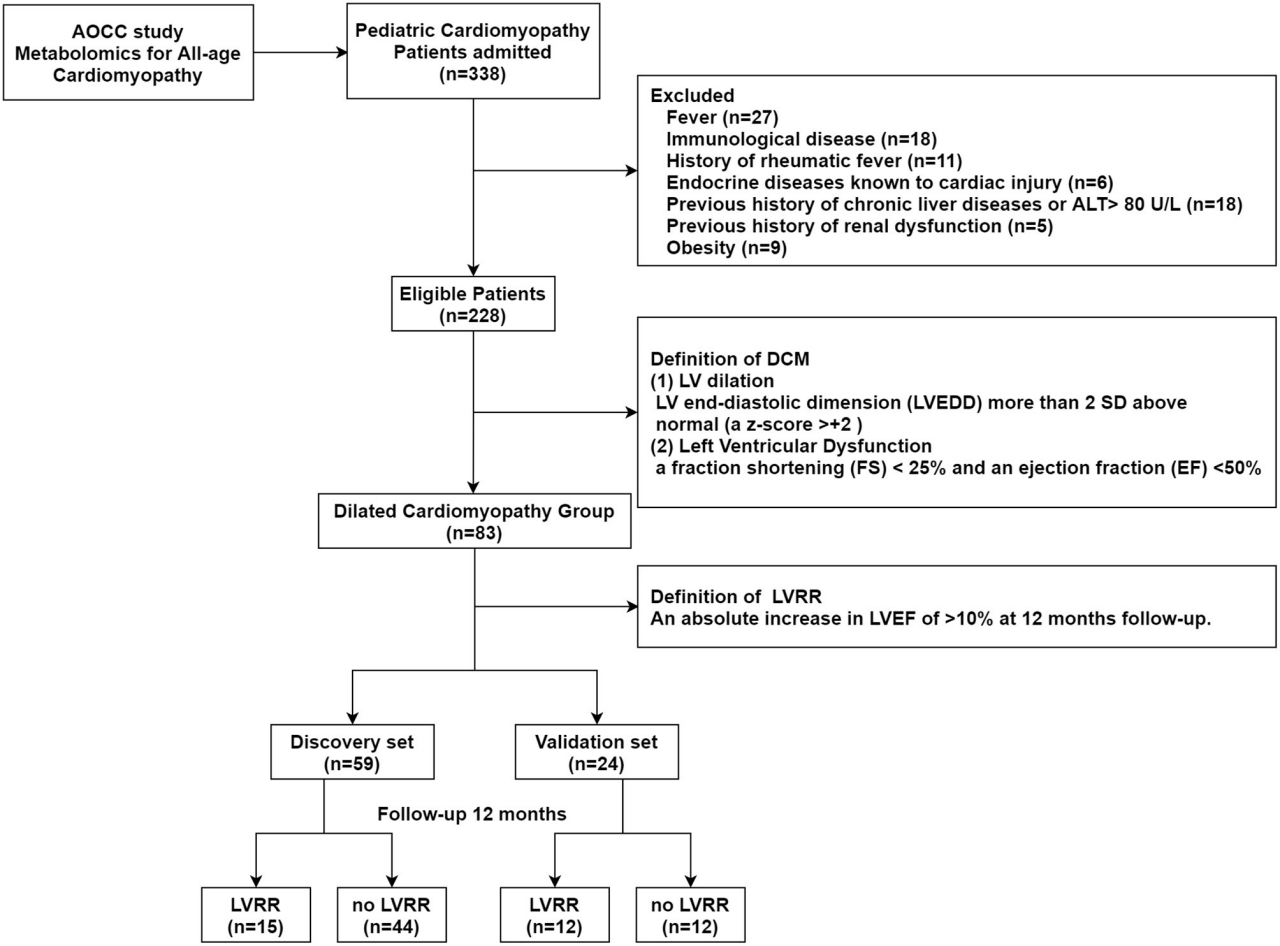
Study flow. The study population enrolled from the China-AOCC (an integrative-omics study of cardiomyopathy patients for diagnosis and prognosis in China) study was divided into discovery and validation sets. LV, left ventricular; LVRR, left ventricular reverse remodeling.

The study is a part of the China-AOCC (An Integrative-Omics Study of Cardiomyopathy Patients for Diagnosis and Prognosis in China) study, a double-center, observational, prospective study. Figure 1 shows the flowchart of the study. The study consisted of a discovery and validation set. For the discovery set, 59 consecutive patients were enrolled between September 2015 and May 2016 in Beijing Anzhen Hospital. The validation set was recruited from July 2016 to March 2017 in Beijing Anzhen Hospital and Beijing Fuwai Hospital.

The inclusion criteria were:

1. Asymptomatic; mild tachypnea, or diaphoresis with feeding in infants; dyspnea on moderate exertion in older children;
2. An age of <18 years;
3. Dilated cardiomyopathy, which was defined as left ventricular or biventricular systolic dysfunction and dilatation not explained by abnormal loading conditions or coronary artery. Systolic dysfunction of DCM, which was defined as abnormal left ventricular ejection fraction (LVEF <50%) and a fraction shortening (FS) of <25%. Left ventricular dilatation, which was defined as LVEDD >2 standard deviations (SDs) from normal according to nomograms (z-scores >2 SDs) corrected by body surface area (BSA) and age. Dilated cardiomyopathy was diagnosed and identified by at least three experienced cardiologists.

The exclusion criteria were:

1. The presence or history of systemic disease, such as diabetes, uremia, rheumatic fever, Kawasaki disease, hypertension, or congenital cardiovascular malformations;
2. The cause of heart muscle disease known to be due to toxic exposure (anthracyclines, mediastinal radiation, iron overload, or heavy metal exposure);
3. Previous history of chronic liver disease or ALT >80 U/L;
4. Previous history of renal dysfunction or creatinine >176 μmol/L;
5. More than 3 months of being bedridden and/or unable to stand alone (age >2 years);
6. Obesity.

The study was designed and carried out by following the principles of the Declaration of Helsinki and was approved by the Beijing Anzhen Hospital and Fuwai Hospital Ethics Committee. Informed consent was obtained from all participants. More details are provided at ClinicalTrials.gov (NCT03076580).

### Definition of Outcome and Follow-Up

The prognostic endpoint was LVRR, an absolute increase in the LVEF (>10%). Patients were followed since the date of DCM diagnosis until the study end date or the end of 1-year follow-up, whichever came first. Follow-up records were based on echocardiographic findings, telephone interviews, and patients' regular visits to outpatient clinics. The outcome was determined by two cardiologists. If there was disagreement about the final diagnosis, a third cardiologist would arbitrate.

### Blood Sample and Clinical Data Collection

Blood samples were collected in the morning following a 12- to 16-h overnight fast and drawn into promoting coagulating tubes. The samples were centrifuged at 3,000 rpm for 10 min in the clinical laboratory. The supernatant serum was quickly removed, aliquoted, and stored at −80°C. All clinical data were extracted and identified from electronic health records, including patients' symptoms, demographics, comorbidities, blood pressures, heart rate, echocardiographic measurements, and laboratory examination.

### Measurement of Lipid Metabolites

Lipid metabolites were extracted from serum samples (20 μl) using a modified Bligh–Dyer extraction process (double extraction), and then we used the appropriate internal standard (16). We scanned lipid metabolites by multiple response monitoring (MRM) on an exion UPLC system (Shimadzu, Kyoto, Japan), and they were analyzed with a 6500 Plus QTRAP (Sciex, Framingham, MA, USA) (17). The missing data rate (patient data and lipid metabolite data) was <5%. Measured lipid metabolite concentrations, denoted as zero, were imputed as half of the minimum reported value. Raw lipid metabolite concentrations were log-transformed and scaled (mean centered and divided by the SD) to maintain a symmetrical and comparable distribution. The false discovery rate (FDR) was used to control for multiple testing. We applied machine learning to identify the most predictive biomarkers. The OPLS-DA model (SIMCA 14.1) and random forest (RF) analysis were used to screen the candidate metabolites of LVRR.

### Statistical Analyses

Continuous variables were presented as median and interquartile range and compared using the Mann–Whitney U-test. Categorical variables are shown as counts with percentages and compared using the chi-square test or the Fisher's exact test. Cox proportional hazards regression was used to calculate unadjusted and adjusted hazard ratios (HRs) after verifying the proportional hazards assumption. Adjusted covariables included age, LVEF, and LVEDD z-score. Spearman's correlation analysis indicated the relationship between candidate lipid metabolites and age. Receiver-operating characteristic (ROC) analysis and Delong test were used to assess the discrimination of the predictive score for LVRR. Kaplan–Meier curves were estimated, and differences between groups were assessed using the log-rank test. Analyses were performed using SPSS 22.0 (IBM, Chicago, IL, USA) and R version 3.4.0 (R Core Team, Vienna, Austria). All tests were two-sided, and a *p*-value of <0.05 was considered statistically significant.

## Results

### Baseline Characteristics

The baseline characteristics of the discovery and validation sets are presented separately in Table 1. Baseline characteristics were compared according to the occurrence of LVRR. In the discovery set, LVRR was found in 15 (25.4%) patients after a median 365 (IQR: 350–365) days of follow-up. The median age of PDCM patients was 19.0 months (IQR: 8.8–58.8) and 26.7% were male. There was no difference in LVEF between the LVRR and no LVRR groups, but LVEDD z-score was lower in the LVRR group upon admission. Laboratory test results and the use of cardiac medications were similar between the LVRR and no LVRR groups. In the validation set, LVRR was found in 12 (50.0%) patients after a median of 341 (IQR: 253–365) days follow-up. The median age of patients with PDCM was 10.0 (5.8–16.8) months, and 41.7% were male. Pediatric dilated cardiomyopathy patients in the LVRR group were younger. Other clinical characteristics were comparable between the two groups. The comparison of LVRR and no LVRR group for all patients is presented in Supplementary Table 1. The levels of total cholesterol and LDL cholesterol in the LVRR group were higher than the no LVRR group. The median levels of total cholesterol in all PDCM patients, LVRR group, and no LVRR group were 4.2, 4.8, and 4.1 mmol/L, respectively. The median levels of LDL cholesterol in all PDCM patients, LVRR group, and no LVRR group were 2.4, 2.9, and 2.3 mmol/L, respectively. However, cholesterol could not predict LVRR (Supplementary Table 2).

**Table 1:** Baseline characteristics of pediatric dilated cardiomyopathy (PDCM) patients in the discovery set and validation set.

**Characteristic**	**Discovery set**				**Validation set**			
	**All**	**LVRR**	**No LVRR**	***p*-Value**	**All**	**LVRR**	**No LVRR**	***p*-Value**
	**(*N* = 59)**	**(*N* = 15)**	**(*N* = 44)**		**(*N* = 24)**	**(*N* = 12)**	**(*N* = 12)**	
**Clinical Characteristics**								
Age, months	22.0 (11.0, 53.5)	19.0 (8.8, 58.8)	26.5 (11.8, 53.3)	0.428	15.0 (8.3, 117.5)	10.0 (5.8, 16.8)	119.0 (15.0, 139.0)	0.039
Sex (male)	22 (37.3)	4 (26.7)	18 (40.9)	0.253	12 (50.0)	5 (41.7)	7 (58.3)	0.342
**Echocardiogram**								
LVEF raw, %	41.0 (30.0, 47.5)	35.0 (28.8, 44.5)	41.5 (30.8, 48.0)	0.329	39.5 (34.5, 44.8)	40.0 (31.0, 47.3)	39.0 (35.0, 42.0)	0.799
LVFS raw, %	20.0 (15.3, 23.0)	20.5 (13.8, 23.0)	20.0 (15.8, 24.0)	0.896	19.5 (16.0, 23.0)	19.0 (13.3, 23.3)	19.0 (17.0, 21.0)	0.755
LVEDD z-score	6.22 (4.18, 9.42)	4.9 (3.1, 7.4)	6.8 (4.7, 10.4)	0.026	5.6 (4.1, 7.2)	4.8 (3.4, 6.8)	7.0 (5.0, 10.8)	0.089
E peak (cm/s)	104.5 (91.5, 115.0)	110.5 (94.5, 130.5)	100.5 (89.5, 109.8)	0.065	105.0 (85.8, 119.3)	109.5 (85.8, 117.5)	106.0 (74.0, 137.0)	1.000
A peak (cm/s)	65.5 (53.0, 83.0)	81.0 (70.3, 85.3)	58.5 (47.0, 80.0)	0.010	71.5 (54.0, 79.5)	70.5 (54.0, 89.5)	71.0 (51.0, 77.0)	0.705
**Laboratory results**								
Glucose, mmol/L	4.9 (4.5, 5.2)	5.1 (4.4, 5.3)	4.9 (4.6, 5.3)	0.556	5.0 (4.5, 5.3)	4.7 (4.4, 5.0)	5.5 (4.6, 5.7)	0.107
Triglycerides, mmol/L	0.8 (0.6, 1.1)	0.7 (0.4, 1.1)	0.8 (0.6, 1.2)	0.246	0.8 (0.6, 1.7)	0.8 (0.7, 1.5)	0.9 (0.5, 1.1)	0.941
LDL cholesterol, mmol/L	2.4 (2.0, 3.0)	2.7 (2.1, 3.1)	2.3 (2.0, 3.0)	0.257	2.5 (1.7, 3.9)	3.3 (1.8, 4.4)	2.0 (1.6, 2.1)	0.230
HDL cholesterol, mmol/L	1.3 (1.1, 1.6)	1.4 (1.1, 1.8)	1.3 (1.0, 1.5)	0.605	1.2 (1.0, 1.6)	1.4 (1.1, 1.7)	1.2 (0.5, 1.6)	0.230
Total cholesterol, mmol/L	4.2 (3.6, 5.1)	4.3 (3.5, 5.9)	4.1 (3.6, 4.7)	0.189	4.5 (3.2, 5.9)	5.3 (3.7, 6.4)	3.1 (2.6, 4.4)	0.056
C-reactive protein, μmol/L	0.2 (0.1, 0.6)	0.2 (0.1, 0.5)	0.2 (0.1, 0.7)	0.717	0.2 (0.1, 0.6)	0.2 (0.1, 0.2)	0.5 (0.2, 12.5)	0.095
Creatinine, μmol/L	30.1 (22.7, 36.9)	28.8 (24.0, 33.9)	28.8 (22.3, 40.3)	0.625	24.2 (16.8, 41.5)	21.8 (16.2, 24.9)	39.6 (25.9, 50.7)	0.017
Urea, μmol/L	4.3 (3.4, 5.3)	4.2 (3.3, 6.0)	4.3 (3.6, 5.4)	0.986	3.7 (3.2, 4.3)	3.4 (2.9, 4.0)	4.1 (3.8, 5.3)	0.030
Sodium, mmol/L	136.9 (135.7, 139.1)	137.6 (136.4, 138.9)	136.5 (135.6, 139.1)	0.536	137.9 (134.8, 138.8)	137.3 (134.0, 138.6)	138.7 (135.7, 139.0)	0.140
BNP, pg/ml	245.0 (65.0, 525.0)	275.0 (96.0, 374.5)	228.0 (71.0, 563.8)	0.917	171.0 (36.5, 468.3)	87.0 (26.8, 236.3)	556.0 (245.0, 1372.0)	0.033
**Cardiac medication**, ***n*****(%)**								
Use of digoxin	57 (96.6)	14 (93.3)	43 (97.7)	0.447	22 (91.7)	12 (100.0)	10 (83.3)	0.239
Use of diuretic agents	52 (88.1)	13 (86.7)	39 (88.6)	0.577	21 (87.5)	12 (100.0)	9 (75.0)	0.109
Use of beta-blocker	28 (47.5)	9 (60.0)	19 (43.2)	0.204	11 (45.8)	6 (50.0)	5 (41.7)	0.500
Use of ACEI	48 (81.4)	12 (80.0)	36 (81.8)	0.574	23 (95.8)	12 (100.0)	11 (91.7)	0.500

*Continuous variables were presented as median and interquartile range and compared using the Mann–Whitney U-test. Categorical variables are shown as counts with percentages and compared using the chi-square test or the Fisher's exact test. LVRR, left ventricular remodeling reverse; LDL, low-density lipoprotein; HDL, high-density lipoprotein; BNP, brain natriuretic peptide; LVEF, left ventricular ejection fraction; LVFS, left ventricular fraction shortening; ACEI, angiotensin-converting enzyme inhibitor*.

### Screening of Candidate Lipid Metabolites

We quantitatively measured 24 lipid classes, 540 lipid species in PDCM patients. The level of each lipid class is shown in Supplementary Table 3. The levels of lysophosphatidic acids (LysoPA), LysoPS, and Free Cho were higher in the LVRR group than in the no LVRR group, but only LysoPA was still significantly higher after FDR correction. The median levels of LysoPA in all PDCM patients, LVRR group, and no LVRR group were 5.74, 10.60, and 4.53 μmol/L, respectively. The OPLS-DA model was used to screen candidate features. There were 248 lipid metabolites with a VIP >1 (Supplementary Table 4). Then, we used RF to define the differences in lipid metabolic profile in the LVRR group and no LVRR group (Supplementary Figure 1). The cross-validation error curve showed that the error rate would increase if the number of features exceeded 20. Thus, the top 20 lipid metabolites were selected (Supplementary Figure 1). The concentrations of the lipid metabolites stratified by LVRR are shown in Supplementary Table 5 with an FDR <0.05. Finally, taking the intersection of the features selected by the three aforementioned methods, we identified four lipid metabolites, LysoPA 16:0, LysoPA 18:2, LysoPA 18:1, and LysoPA 18:0.

### LysoPA Is Independent Predictor of Left Ventricular Reverse Remodeling

In the discovery set, LysoPA 16:0, LysoPA 18:2, LysoPA 18:1, and LysoPA 18:0 were significantly associated with LVRR. The HRs were 2.576 (95% CI 1.507–4.403), 3.094 (95% CI 1.720–5.568), 2.956 (95% CI 1.650–5.294), and 2.654 (95% CI 1.529–4.610), respectively (Table 2). After adjusting for age, LVEF, and LVEDD z-score, the HRs were 2.793 (95% CI 1.545–5.048), 2.812 (95% CI 1.542–5.128), 2.831 (95% CI 1.555–5.154), and 2.782 (95% CI 1.548–5.002), respectively. The results were consistent in the validation set (Table 2). We also investigated the correlation between LysoPA and age (Supplementary Figure 2). There was no significant correlation between LysoPA and age. Our results indicated that four LysoPAs were independent predictors of LVRR.

**Table 2:** Association between LysoPA and LVRR in the discovery set and validation set.

	**Univariate model^a^**		**Multivariable model^a^**		**Univariate model^b^**		**Multivariable model^b^**	
	**Hazard ratio (95% CI)**	***p*-Value**	**Hazard ratio (95% CI)**	***p*-Value**	**Hazard ratio (95% CI)**	***p*-Value**	**Hazard ratio (95% CI)**	***p*-Value**
LysoPA 16:0	2.576 (1.507–4.403)	0.001	2.793 (1.545–5.048)	0.001	3.626 (1.564–8.409)	0.003	5.100 (1.637–15.891)	0.005
LysoPA 18:2	3.094 (1.720–5.568)	<0.001	2.812 (1.542–5.128)	0.001	4.302 (1.656–11.176)	0.003	6.777 (1.713–26.809)	0.006
LysoPA 18:1	2.956 (1.650–5.294)	<0.001	2.831 (1.555–5.154)	0.001	4.232 (1.668–10.737)	0.002	6.254 (1.782–21.955)	0.004
LysoPA 18:0	2.654 (1.529–4.610)	0.001	2.782 (1.548–5.002)	0.001	4.812 (1.788–12.950)	0.002	9.605 (2.23 – -41.334)	0.002

*Weighted Cox regression was performed to identify LysoPA subclasses associated with LVRR. The model was adjusted for age, LVEF, and LVEDD z-score. LVRR, left ventricular reverse remodeling; LVEDD, left ventricular end-diastolic dimension*.

a *Association between LysoPA and LVRR in the discovery set*.

b *Association between LysoPA and LVRR in the validation set*.

### LysoPA Score and Prediction for Left Ventricular Reverse Remodeling

**Figure 2:**
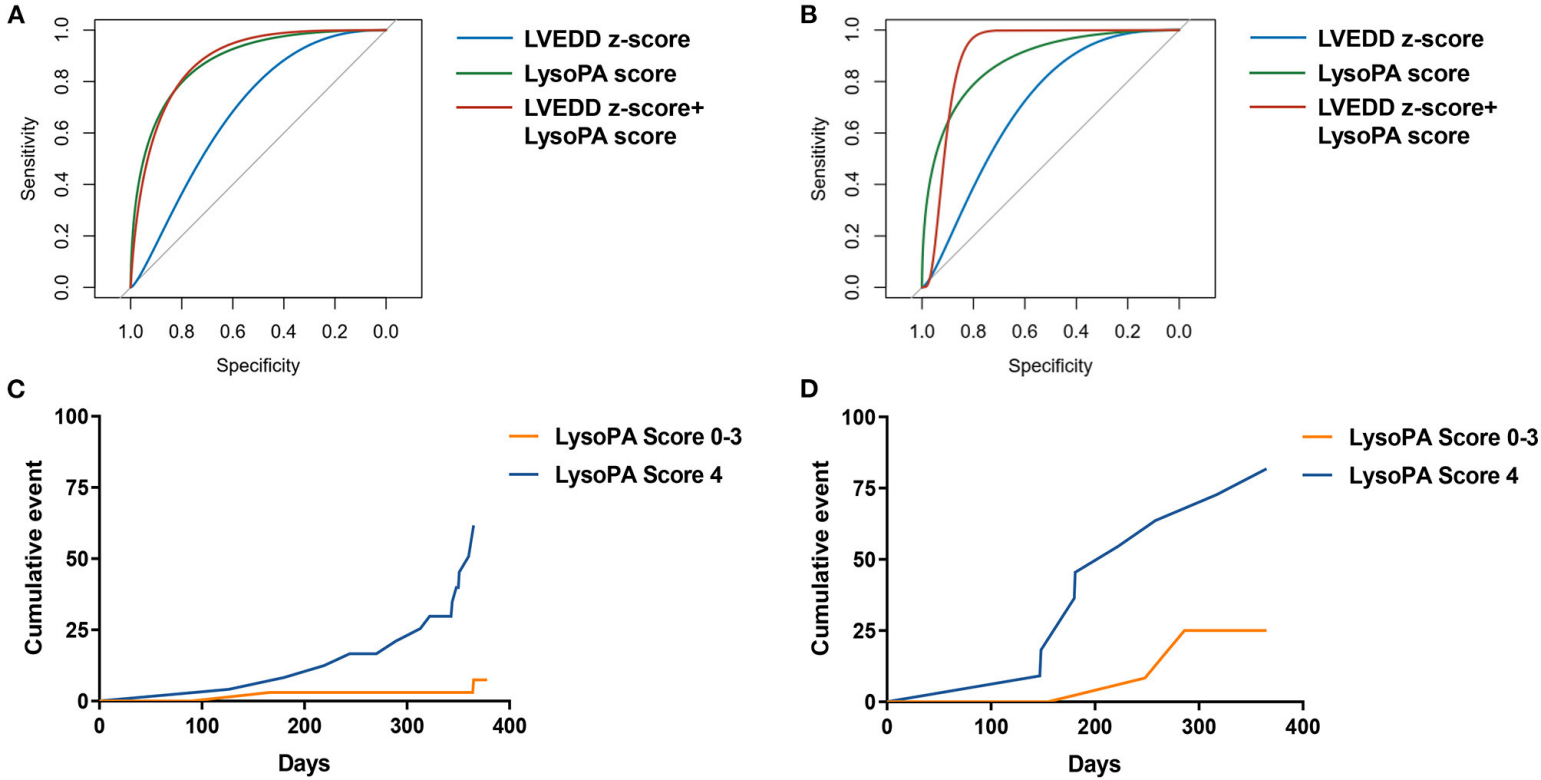
The prediction of LysoPA score for LVRR. ROC curves for predicting LVRR in **(A)** discovery set and **(B)** validation set. Kaplan–Meier cumulative event curve of LysoPA score for predicting LVRR in **(C)** the discovery set and **(D)** validation set.

**Figure 3:**
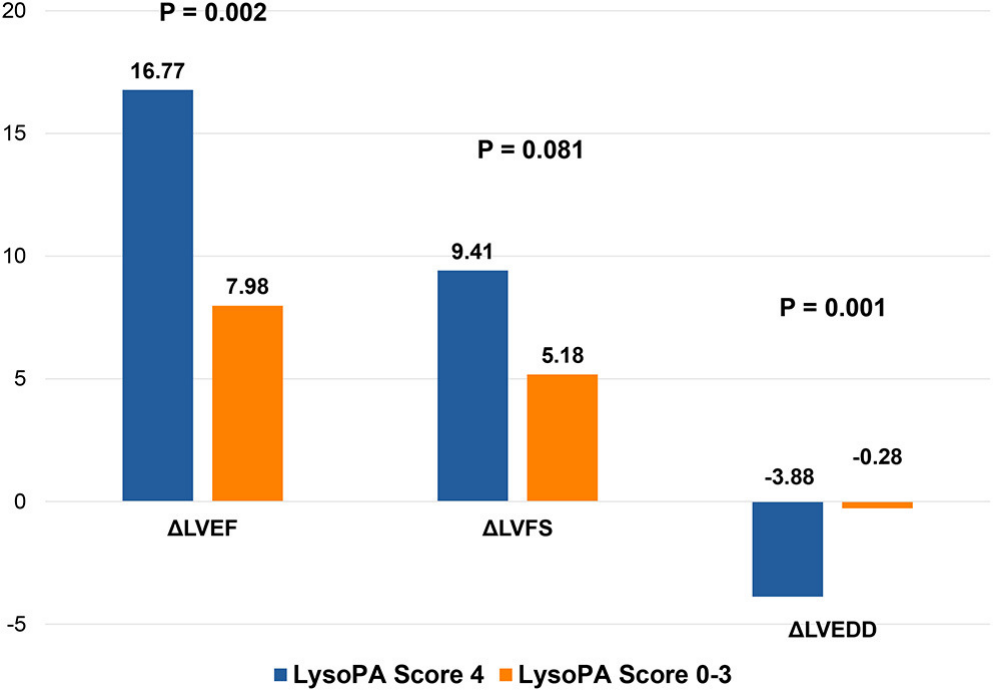
Changes in left ventricular structure and function in different LysoPA score group during 1 year. LVEF, left ventricular ejection fraction; LVFS, left ventricular fraction shortening; LVEDD, left ventricular end-diastolic dimension.

We set up a LysoPA score based on four LysoPA subclasses to transform the clinical application of these LVRR predictors. If the variable was above the median, the individual received one point. Thus, the score ranged from 0 to 4. Based on the score, the individuals were split into two risk categories (LysoPA score = 0–3 and LysoPA score = 4). The incidence of LVRR was higher when the LysoPA score reached 4, both in the discovery set (54.2 vs. 5.7%; relative risk: 9.5) and validation set (81.8 vs. 23.1%; relative risk: 3.5) (Table 3). Figure 2 shows the prediction of LysoPA score for LVRR in the discovery set and validation set. After combining the discovery and validation sets, we found that the improvements of left ventricular function indicators from baseline to 1-year follow-up were more significant when the LysoPA score reached 4 (Figure 3).

**Table 3:** LysoPA score and prediction for LVRR.

**Discovery set**					**Validation set**				
**LysoPA score**	**LVRR**	**No LVRR**	**LVRR %**	**Relative Risk**	**LysoPA Score**	**LVRR**	**No LVRR**	**LVRR %**	**Relative Risk**
0–3	2	33	5.7	As reference	0–3	3	10	23.1	As reference
4	13	11	54.2	9.5 (2.3–38.3)	4	9	2	81.8	3.5 (1.3–9.9)

*If the concentration of the LysoPA subclass was above the median, the individual received one point. Thus, the score ranged from 0 to 4. LVRR, left ventricular reverse remodeling*.

### LysoPA Score Improves the Performance of Left Ventricular End-Diastolic Dimension Z-Score

Pediatric dilated cardiomyopathy patients can recover to normal LV size and function, particularly those with a smaller LVEDD z-score at diagnosis (9). We determined whether the LysoPA score could improve the LVEDD z-score for predicting the occurrence of LVRR. The AUC increased with the addition of LysoPA score to LVEDD z-score from 0.693 (95% CI 0.560–0.807, *p* = 0.009) to 0.875 (95% CI 0.763–0.947, *p* < 0.001) for the prediction of LVRR, and the ΔAUC was 0.182 (95% CI 0.053–0.310, *p* = 0.006) in the discovery set (Table 4). In the validation set, the AUC also increased with the addition of LysoPA score to LVEDD z-score from 0.708 (95% CI 0.489–0.874, *p* = 0.063) to 0.854 (95% CI 0.651–0.964, *p* < 0.001) for the prediction of LVRR, and the ΔAUC was 0.146 (95% CI −0.114–0.406, *p* = 0.271) (Table 4).

**Table 4:** Prediction of LysoPA score and LVEDD z-score for LVRR in the discovery set and validation set.

	**Discovery set**				**Validation set**			
**Model**	**AUC**	***p*-Value**	**ΔAUC**	***p*-Value**	**AUC**	***p*-Value**	**ΔAUC**	***p*-Value**
	**(95% CI)**		**(95% CI)**		**(95% CI)**		**(95% CI)**	
LVEDD z-score	0.693 (0.560, 0.807)	0.009			0.708 (0.489, 0.874)	0.063		
LysoPA score	0.820 (0.698, 0.908)	<0.001	0.127 (−0.047, 0.300)	0.154	0.816 (0.606, 0.943)	<0.001	0.108 (−0.178, 0.393)	0.460
LVEDD z-score + LysoPA score	0.875 (0.763, 0.947)	<0.001	0.182 (0.053, 0.310)	0.006	0.854 (0.651, 0.964)	<0.001	0.146 (−0.114, 0.406)	0.271

*LVRR, left ventricular reverse remodeling; LVEDD, left ventricular end-diastolic dimension*.

**Figure 4:**
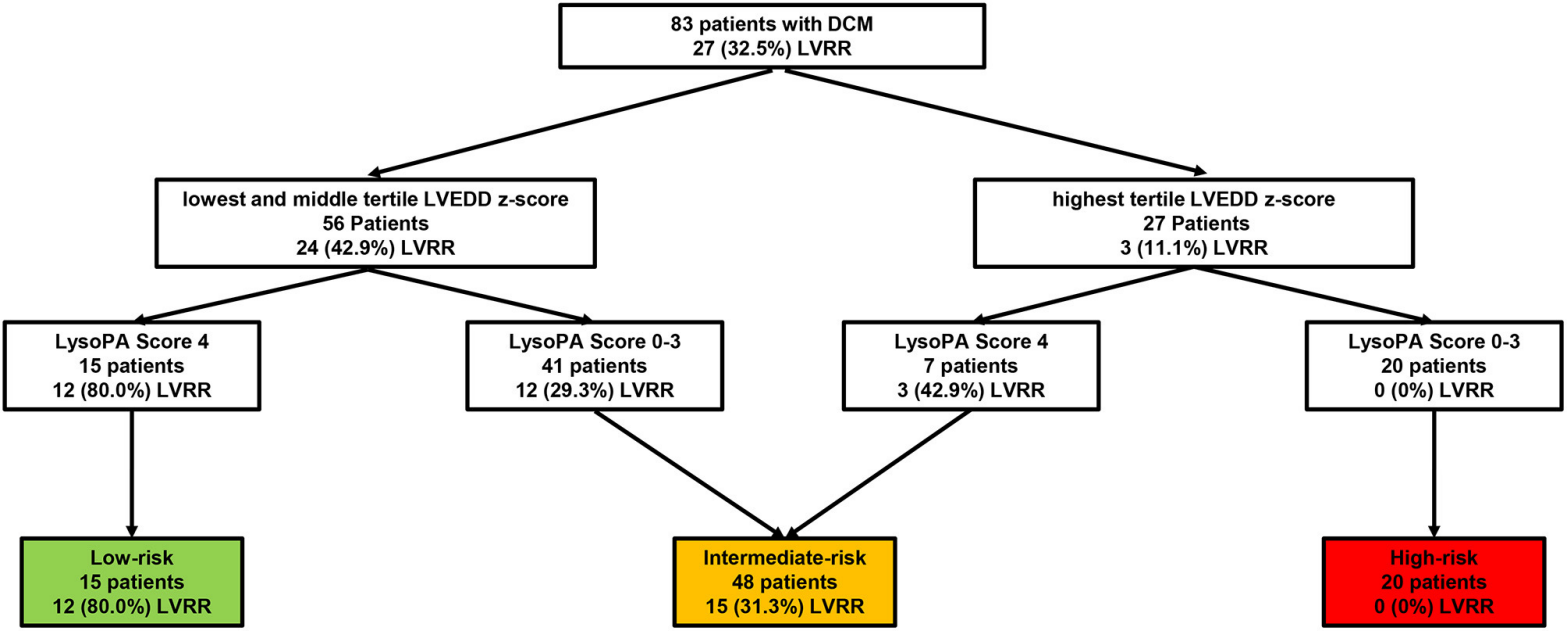
Risk assessment strategy of patients with PDCM. The number (%) of patients with LVRR is shown for each step. Cutoffs were applied at LVEDD z-score in the highest tertile and LysoPA score = 4. PDCM, pediatric dilated cardiomyopathy; LVRR, left ventricular reverse remodeling; LVEDD, left ventricular end-diastolic dimension.

We stratified patients by LVEDD z-score and LysoPA score. The cutoff of LVEDD z-score was the highest tertile (18). Thus, patients were divided into the low-risk and high-risk groups by the LVEDD z-score. Meanwhile, by adding the LysoPA score, we were able to differentiate patients with and without LVRR better (Figure 4).

## Discussion

To the best of our knowledge, this is the first report on the prediction of cardiac function recovery in PDCM using lipid metabolites. First, serum LysoPA level was the most significant predictor of LVRR, which was independent of other lipid metabolites. Additionally, we set up a LysoPA score composed of four LysoPA metabolites. The LysoPA score can predict the occurrence of LVRR. Finally, combining the LysoPA score with the LVEDD z-score could improve the risk stratification of PDCM.

Even though 30–50% of pediatric patients are classified as familial DCM due to genetic factors, abnormal lipid metabolism is also a major pathogenic factor (19). We identified four LysoPA metabolites, which were the most prevalent features in human plasma, as 18:2 (50%), 18:1 (15%), 18:0 (13%), and 16:0 (12%), respectively (20), suggesting that these LysoPA subclasses are likely to play an important biological role as the major components of LysoPA. Lysophosphatidic acids is released from activated cells, such as platelets, leukocytes, and fibroblasts (21), which acts via six kinds of G protein-coupled receptors (GPCRs)–LPA1-6 on the cell surface to activate a great variety of signaling pathways (22). The principal effects of LysoPA are stimulation of cell growth, prevention of apoptosis, regulation of actin cytoskeleton, and modulation of cell shape (23).

There has been no report on the association between lipid profile and clinical outcomes in the pediatric DCM cohort. Sysi-Aho et al. discovered that serum triglycerides were associated with clinical adverse events, as well as cardiac structure and volume changes in an adult DCM cohort (24). In our study, the levels of total cholesterol were higher in the LVRR group than in the no LVRR group (4.8 vs. 4.1 mmol/L); however, the cholesterol levels among PDCM patients were generally within the normal ranges. Dyslipidemia was defined by elevated levels of LDL-C (130 or 3.37 mmol/L) or total cholesterol (200 or 5.18 mmol/L) (25). Furthermore, there was no significant correlation between total cholesterol and LVRR (Supplementary Table 2). Lysophosphatidic acids may play a protective role for the following reasons. First, LysoPA may prevent mitochondria-related myocardial fibrosis and necrosis by promoting the normal fission of mitochondria in DCM. Song et al. (26) reported that cardiomyocyte-specific Drp1 ablation evoked mitochondrial enlargement, lethal DCM, and cardiomyocyte necrosis due to the interruption of mitochondrial fission. Meanwhile, LysoPA is synthesized on the mitochondrial outer membrane (27) and may have a pro-fission function when elevated in concentration on the surface of the mitochondria (28). Lysophosphatidic acids almost completely blocked mitochondria-dependent apoptosis in mesenchymal stem cells after myocardial infarction via the regulation of the p38 pathway by LPA(1/3)/Gi/ERK1/2 pathway-mediated MKP-1 induction (29). Second, a previous study reported that LysoPA preconditioning could also prevent oxidative stress-induced cardiomyocyte apoptosis and increase glucose uptake, finally improving the functional recovery of infantile hearts after ischemia reperfusion, which was mediated through the LysoPA receptor 1 and/or 3/AKT/GSK3β pathways (30). Finally, LysoPA has been shown to be protective in inflammation. Murch et al. (31) found that therapeutic administration of LysoPA 18:0 or 18:1 could reduce the organ injury caused by LPS, and LysoPA 18:0 also attenuated the LPS-induced increase in plasma IL-1beta levels. Nevertheless, some contrarious evidence indicates that LysoPA not only leads to cardiomyocyte apoptosis (32) but also activates MMP and induces fibrosis through the NF-kB pathway (33). Therefore, the effects of LysoPA on cardiomyocytes are controversial, prompting further verification.

We set up a LysoPA score and found that it could identify 80% of PDCM with improved cardiac function after 1 year. The effect of risk stratification by LysoPA score combined with LVEDD z-score was better than using LVEDD z-score alone. On the one hand, we make a step forward in distinguishing patients with different outcomes. On the other hand, this also helped in monitoring the effects of anti-heart failure pharmacotherapy.

This study had some limitations. First, the sample size was small, which was limited by disease incidence. Although all PDCM patients were enrolled consecutively, 15/59 (25%) of the patients manifested LVRR in the discovery set, whereas 12/24 (50%) manifested LVRR in the validation set. This might be due to the low sample size and the difference in age between the LVRR and no LVRR group in the validation set. As previously reported by Fenton MJ (9), younger age was one of the predictors of recovery in PDCM patients. Therefore, the findings cannot be generalized to the entire PDCM population. Second, although the discharge medication was similar in the LVRR and no LVRR groups, we did not consider treatment compliance during follow-up. Third, we did not perform a genetic test, so we could not differentiate the pathogenesis at the genetic level. Fourth, the use of beta-blocker in our PDCM dataset was relatively low compared with other countries. Finally, we did not verify the results of this study in an external cohort.

## Conclusion

In conclusion, this is the first study to show the relationship between serum lipidomics profiles and LVRR in PDCM. Serum LysoPA can predict LVRR in PDCM. When the LysoPA score was combined with the LVEDD z-score, it could help for ascertaining the prognosis and monitoring effects of anti-heart failure pharmacotherapy.

## Data Availability Statement

The original contributions presented in the study are included in the article/Supplementary Material, further inquiries can be directed to the corresponding author/s.

## Ethics Statement

The studies involving human participants were reviewed and approved by ethics committee of Beijing anzhen hospital capital medical university. Written informed consent to participate in this study was provided by the participants' legal guardian/next of kin. Written informed consent was obtained from the minor(s)' legal guardian/next of kin for the publication of any potentially identifiable images or data included in this article.

## Author Contributions

HW designed the study, coordinated data collection, carried out the analyses, and drafted the initial manuscript. HY conceptualized and designed the study, critically reviewed, and revised the manuscript. YL and KM coordinated data collection, carried out the analyses, and revised the manuscript. SW carried out the analyses and revised the manuscript. MJia, SY, JH, JS, YG, ZW, and PZ coordinated the data, blood sample collection, reviewed, and revised the manuscript. YW, MJin, and JD participated in the study design, carried out the analyses, reviewed, and revised the manuscript. All authors approved the final manuscript as submitted and agreed to be accountable for all aspects of the work.

## Conflict of Interest

The authors declare that the research was conducted in the absence of any commercial or financial relationships that could be construed as a potential conflict of interest.

## Publisher's Note

All claims expressed in this article are solely those of the authors and do not necessarily represent those of their affiliated organizations, or those of the publisher, the editors and the reviewers. Any product that may be evaluated in this article, or claim that may be made by its manufacturer, is not guaranteed or endorsed by the publisher.

## Abbreviations

LysoPA: lysophosphatidic acids
PDCM: pediatric dilated cardiomyopathy
LVRR: left ventricular reverse remodeling
LVEDD: left ventricular end-diastolic dimension
DCM: dilated cardiomyopathy
LVEF: left ventricular ejection fraction
FS: fraction shortening
BSA: body surface area
RF: random forest.
