# Supplementary material for: Higher Serum Lysophosphatidic Acids Predict Left Ventricular Reverse Remodeling in Pediatric Dilated Cardiomyopathy
Source: Front Pediatr. 2021 Aug 16;9:710720. doi: 10.3389/fped.2021.710720 (PMC8415784; doi:10.3389/fped.2021.710720)
Supplement: Supplementary file 1 [file Data_Sheet_1.docx]

Supplemental tables

Supplemental table 1. Baseline Characteristics of PDCM patients from China-AOCC study

| **Characteristic** | **All**  **(n=83)** | **LVRR**  **(n=27)** | **NoLVRR**  **(n=56)** | **P** |
| --- | --- | --- | --- | --- |
| **Clinical characteristics** | | | | |
| Age, Months | 21.0 (10.0, 55.0) | 12.0 (8.0,22.0) | 28.5 (12.3, 67.5) | 0.015 |
| Sex (Male) | 34 (41.0) | 9 (33.3) | 25 (44.6) | 0.352 |
| **Echocardiogram** | | | | |
| LVEF raw, % | 40.5(31.0, 47.0) | 38.0(30.5, 46.5) | 41.0(33.5, 47.5) | 0.477 |
| LVFS raw, % | 20.0 (16.0, 23.0) | 20.0 (14.0,23.0) | 20.0 (17.0, 23.0) | 0.661 |
| LVEDD z-score | 6.2(4.4, 9.0) | 5.0(3.3, 7.0) | 6.8(4.9, 10.4) | 0.006 |
| E peak (cm/s) | 103.0(90.8, 116.3) | 110.0(94.0, 121.5) | 101.0(89.0, 113.5) | 0.104 |
| A peak (cm/s) | 66.5(52.8, 82.3) | 74.0(65.5, 85.0) | 59.0(49.0, 78.5) | 0.032 |
| **Laboratory results** | | | | |
| Glucose, mmol/L | 5.0(4.5, 5.3) | 5.0(4.5, 5.2) | 5.0 (4.6, 5.3) | 0.645 |
| Triglycerides, mmol/L | 0.8 (0.6,1.2) | 0.8 (0.5,1.3) | 0.8 (0.6,1.1) | 0.474 |
| LDL cholesterol, mmol/L | 2.4 (2.0,3.2) | 2.9 (2.1,3.8) | 2.3 (1.9,3.0) | 0.046 |
| HDL cholesterol, mmol/L | 1.3(1.1, 1.6) | 1.4(1.1, 1.7) | 1.3(1.0, 1.5) | 0.296 |
| Total cholesterol, mmol/L | 4.2 (3.5,5.3) | 4.8 (3.7,5.9) | 4.1 (3.4,4.7) | 0.022 |
| C-reactive protein, μmol/L | 0.2 (0.1,0.6) | 0.2 (0.1,0.3) | 0.3 (0.1,1.1) | 0.183 |
| Creatinine, μmol/L | 27.6 (22.4,39.1) | 24.6 (20.9,33.5) | 29.8 (22.5,41.1) | 0.034 |
| Urea, μmol/L | 4.0 (3.3,5.2) | 3.4 (2.9,4.6) | 4.2 (3.6.5.3) | 0.090 |
| Sodium, mmol/L | 136.8(135.7, 138.9) | 137.1(135.6, 138.7) | 136.7(135.7, 139.0) | 0.845 |
| BNP, pg/mL | 245.0(68.8, 529.5) | 147.0(62.5, 341.0) | 271.0(84.0, 606.0) | 0.172 |
| **Cardiac medication, n (%)** | | | | |
| Use of digoxin | 79 (95.2) | 26 (96.3) | 53 (94.6) | 1.000 |
| Use of diuretic agents | 73 (88.0) | 25 (92.6) | 48 (85.7) | 0.487 |
| Use of beta-blocker | 39 (47.0) | 15 (55.6) | 24 (42.9) | 0.350 |
| Use of ACEI | 71 (85.5) | 24 (88.9) | 47 (83.9) | 0.743 |

Continuous variables were presented as median and interquartile range and compared using the Mann-Whitney U test. Categorical variables are shown as counts with percentages and compared using the chi-square test or the Fisher’s exact test. Abbreviations: LVRR, left ventricular remodeling reverse; LDL, low-density lipoprotein; HDL, high-density lipoprotein; BNP, brain natriuretic peptide; LVEF, left ventricular ejection fraction; LVFS, left ventricular fraction shortening; ACEI, Angiotensin-Converting Enzyme Inhibitor.

Supplemental table 2. Association between clinical covariates and LVRR in the discovery set and validation set.

|  | *Discovery set* | |  | *Validation set* | |
| --- | --- | --- | --- | --- | --- |
| clinical covariates | Hazard ratio (95% CI) | P |  | Hazard ratio (95% CI) | P |
| Age | 0.995 (0.981 - 1.009) | 0.492 |  | 0.981 (0.962 - 1.000) | 0.048 |
| LVEF | 0.964 (0.906 - 1.024) | 0.234 |  | 1.012 (0.929 - 1.103) | 0.778 |
| LVEDD z-score | 0.837 (0.694 - 1.010) | 0.064 |  | 0.851 (0.675 - 1.072) | 0.172 |
| Triglyceride | 0.511 (0.171-1.530) | 0.230 |  | 1.541 (0.396 - 5.991) | 0.532 |
| LDL cholesterol | 1.251 (0.739 - 2.119) | 0.404 |  | 1.110 (0.879 - 1.401) | 0.381 |
| HDL cholesterol | 2.483 (0.821 - 7.509) | 0.107 |  | 2.962 (0.737 - 11.900) | 0.126 |
| Total cholesterol | 1.400 (0.890 - 2.201) | 0.145 |  | 1.116 (0.921 - 1.352) | 0.262 |

Supplemental table 3. Medians and interquartile ranges of lipid classes in LVRR group and no LVRR group.

| Lipid class | All | LVRR | no LVRR | P | FDR |
| --- | --- | --- | --- | --- | --- |
| LysoPA | 5.74 (3.46, 10.40) | 10.60 (8.04, 18.10) | 4.53 (3.07, 7.05) | <0.001 | 0.002 |
| LysoPC | 172.77 (162.10, 196.17) | 186.49 (154.20, 202.06) | 172.14 (162.62, 189.27) | 0.243 | 0.395 |
| LysoPE | 23.80 (18.90, 34.60) | 23.30 (16.90, 36.90) | 24.45 (19.68, 34.40) | 0.889 | 0.963 |
| LysoPI | 1.96 (1.47, 2.33) | 1.96 (1.57, 3.74) | 1.93 (1.34, 2.33) | 0.449 | 0.687 |
| LysoPS | 0.47 (0.31, 0.82) | 0.86 (0.46, 1.12) | 0.42 (0.29, 0.66) | 0.005 | 0.043 |
| TAG | 909.06 (627.97, 1179.02) | 1103.52 (626.85, 1316.62) | 890.9 (636.54, 1116.78) | 0.589 | 0.806 |
| DAG | 24.30 (18.80, 33.60) | 31.30 (23.50, 35.90) | 23.00 (18.50, 31.08) | 0.123 | 0.320 |
| CE | 647.78 (579.94, 746.78) | 691.08 (608.70, 770.36) | 642.21 (560.50, 723.84) | 0.091 | 0.309 |
| Free Cho | 1491.49 (1263.55, 1754.99) | 1595.77 (1505.21, 1833.12) | 1420.46 (1195.57, 1665.48) | 0.043 | 0.265 |
| keto-Cho | 0.19 (0.17, 0.22) | 0.18 (0.17, 0.21) | 0.19 (0.16, 0.22) | 0.589 | 0.806 |
| FFA | 163.61 (127.74, 218.89) | 201.87 (142.90, 224.37) | 156.00 (122.56, 213.05) | 0.198 | 0.395 |
| PC | 1393.55 (1207.03, 1564.16) | 1369.99 (1250.57, 1584.82) | 1401.21 (1174.13, 1559.85) | 0.794 | 0.912 |
| PE | 74.60 (58.60, 89.80) | 84.20 (68.50, 89.80) | 73.15 (55.43, 90.58) | 0.204 | 0.395 |
| PA | 0.11 (0.09, 0.13) | 0.11 (0.09, 0.13) | 0.10 (0.09, 0.13) | 0.807 | 0.912 |
| PS | 0.69 (0.57, 0.80) | 0.72 (0.68, 0.95) | 0.66 (0.56, 0.78) | 0.223 | 0.395 |
| PI | 56.90 (47.80, 67.20) | 56.90 (47.80, 67.00) | 56.85 (46.90, 67.98) | 1.000 | 1.000 |
| PG | 0.88 (0.76, 1.20) | 1.00 (0.63, 1.25) | 0.87 (0.76, 1.20) | 0.794 | 0.912 |
| SM | 392.05 (339.22, 479.57) | 432.96 (377.14, 523.98) | 384.90 (337.70, 440.38) | 0.051 | 0.265 |
| GM3 | 6.82 (5.73, 8.74) | 7.63 (6.59, 10.00) | 6.64 (5.62, 8.08) | 0.068 | 0.295 |
| LBPA | 0.35 (0.23, 0.76) | 0.25 (0.20, 0.42) | 0.37 (0.24, 0.81) | 0.096 | 0.309 |
| Cer | 5.16 (4.30, 6.14) | 5.09 (4.59, 6.51) | 5.19 (4.26, 6.10) | 0.638 | 0.829 |
| GluCer | 4.13 (3.19, 4.70) | 4.21 (3.25, 5.52) | 4.01 (3.15, 4.63) | 0.237 | 0.395 |
| LacCer | 1.28 (1.02, 1.53) | 1.24 (1.03, 1.64) | 1.28 (1.00, 1.52) | 0.979 | 1.000 |
| S1P | 0.62 (0.54, 0.73) | 0.73 (0.54, 0.81) | 0.61 (0.52, 0.69) | 0.107 | 0.309 |

The unit of the level of lipid class is μmol/L.

CE, cholesteryl esters; Cer, Ceramides; DAG, diacylglycerols; Free Cho, Free Cholesterol; FFA, Free fatty acids;GluCer, Glucosylceramides; GM3, Dihexosyl-monosialo-gangliosides; keto-Cho, Keto-cholesterol; LacCer, Lactosylceramides; LBPA, Lyso-bisphophatidic acids; LysoPA, Lyso-phosphatidicacids; LysoPC, Lyso-phosphatidylcholines; LysoPE, Lyso-phosphatidylethanolamines; LysoPI, Lyso-phosphatidylinostiols; LysoPS, Lyso-phosphatidylserines; PA, Phosphatidic acids; PC, Phosphatidylcholines; PE, Phosphatidylethanolamines; PG, Phosphatidylglycerols; PI, Phosphatidylinostiols; PS, Phosphatidylserines; S1P, Shingosine-1-phosphates; SM, Sphingomyelins; TAG, triacylglycerols.

|  |
| --- |
|  |
|  |
|  |

Supplemental table 4. Lipid metabolites with VIP value >1 in the OPLS-DA model.

| Lipid metabolite | VIP |
| --- | --- |
| LysoPC22:6 | 1.8618 |
| PE40:6p(22:6) | 1.83037 |
| PE40:6p | 1.73552 |
| PC36:4(20:3) | 1.73141 |
| CE22:6 | 1.72318 |
| CE22:5 | 1.68195 |
| TAG51:2(17:1) | 1.65037 |
| TAG56:8(18:2) | 1.63534 |
| TAG50:2(16:1) | 1.63248 |
| TAG52:2(16:1) | 1.6293 |
| TAG50:1(16:1) | 1.62504 |
| TAG51:0(17:0) | 1.62227 |
| FA22:6 | 1.61256 |
| PC34:3(16:1) | 1.60394 |
| TAG50:2(18:1) | 1.5826 |
| TAG52:1(16:1) | 1.57638 |
| PC38:4(20:3) | 1.55772 |
| GM3 18:0/16:0 | 1.55745 |
| TAG56:8(22:6) | 1.55581 |
| PC38:6 | 1.54303 |
| TAG48:1(16:0) | 1.53985 |
| PE38:6p | 1.53803 |
| TAG50:1(18:0) | 1.53391 |
| TAG58:9(22:6) | 1.53264 |
| TAG58:9(22:5) | 1.52552 |
| TAG48:2(16:1) | 1.52485 |
| SM18:0/16:0 | 1.51798 |
| FA20:4 | 1.51742 |
| PE34:3(16:1) | 1.50483 |
| TAG58:8(22:5) | 1.50149 |
| TAG52:3(16:1) | 1.49563 |
| LPA18:2 | 1.48719 |
| TAG48:1(16:1) | 1.48654 |
| PE36:4p | 1.48166 |
| TAG56:7(18:2) | 1.47748 |
| TAG53:2(19:1) | 1.47407 |
| CE22:2 | 1.47028 |
| TAG48:0(16:0) | 1.45134 |
| PI38:5(22:5) | 1.45103 |
| PC32:1 | 1.45004 |
| PE34:1 | 1.44813 |
| TAG51:2(15:0) | 1.44671 |
| SM18:1/16:0 | 1.43381 |
| TAG50:0(18:0) | 1.43067 |
| PC40:6(20:3) | 1.42155 |
| TAG50:1(16:0) | 1.41998 |
| TAG50:1(18:1) | 1.4185 |
| PC38:6(20:3) | 1.41762 |
| TAG52:0(18:0) | 1.41678 |
| PC38:5(20:3) | 1.4113 |
| TAG53:3(17:1) | 1.41107 |
| TAG56:7(22:5) | 1.41035 |
| PE40:5(22:5) | 1.40971 |
| TAG54:3(16:1) | 1.40801 |
| PC40:3p(20:3) | 1.40645 |
| PE38:6p(22:6) | 1.40156 |
| SM18:1/18:0 | 1.39561 |
| FA22:5 | 1.3908 |
| CE20:4 | 1.38473 |
| PE36:3 | 1.38386 |
| PC38:5(22:4) | 1.37978 |
| FA18:2 | 1.37744 |
| TAG56:6(18:2) | 1.36802 |
| PI38:5(20:5) | 1.36543 |
| CE18:2 | 1.36126 |
| PG38:4 | 1.36052 |
| PI34:1 | 1.35906 |
| PE32:1 | 1.35765 |
| TAG56:7(20:4) | 1.35732 |
| TAG53:2(17:0) | 1.3492 |
| PG36:4(20:4) | 1.34706 |
| GM3 18:1/16:0 | 1.34358 |
| TAG52:2(18:1) | 1.34049 |
| PC40:6(22:5) | 1.33826 |
| PC38:6(22:5) | 1.33257 |
| PI38:5 | 1.33149 |
| TAG48:1(18:1) | 1.32112 |
| TAG52:1(18:1) | 1.32002 |
| LPA16:0 | 1.31948 |
| PE38:5p | 1.31318 |
| CE20:1 | 1.30601 |
| LBPA36:2 | 1.30601 |
| PG38:3 | 1.30519 |
| DAG38:4(18:0/20:4) | 1.3045 |
| TAG48:2(16:0) | 1.30287 |
| TAG54:0(18:0) | 1.29835 |
| TAG51:3(17:1) | 1.29689 |
| LPA18:1 | 1.29583 |
| TAG48:3(16:0) | 1.29566 |
| LPA18:0 | 1.29541 |
| TAG52:2(16:0) | 1.29261 |
| TAG50:3(16:2) | 1.29255 |
| DAG36:4(18:2/18:2) | 1.28961 |
| TAG56:6(20:2) | 1.28337 |
| TAG54:1(18:1) | 1.28138 |
| PE38:5(22:5) | 1.27736 |
| TAG54:1(18:0) | 1.27582 |
| TAG51:2(17:0) | 1.27059 |
| LBPA36:3 | 1.26981 |
| PE36:3(18:1) | 1.26477 |
| PC36:5(20:4) | 1.26469 |
| LysoPC20:4 | 1.26038 |
| PC34:3 | 1.25876 |
| PE36:2(18:0) | 1.25871 |
| TAG56:6(22:4) | 1.25747 |
| TAG50:3(16:1) | 1.25624 |
| PC36:3(20:3) | 1.2529 |
| TAG51:3(15:1) | 1.25254 |
| PC40:6 | 1.25124 |
| PC40:5(20:3) | 1.24594 |
| TAG50:2(16:0) | 1.24492 |
| TAG54:4(16:1) | 1.24423 |
| PG38:6 | 1.24383 |
| DAG34:2(16:0/18:2) | 1.23601 |
| PG36:3 | 1.2357 |
| TAG50:3(16:0) | 1.23302 |
| TAG52:4(16:0) | 1.23256 |
| SM18:0/24:0 | 1.23085 |
| SM18:1/24:0 | 1.2306 |
| LBPA36:1 | 1.22735 |
| TAG50:3(18:2) | 1.22637 |
| PE36:4(18:1) | 1.22549 |
| TAG56:7(22:6) | 1.22442 |
| CE20:3 | 1.22351 |
| TAG52:4(18:2) | 1.22052 |
| TAG48:3(16:1) | 1.21932 |
| PE36:3p | 1.20827 |
| TAG54:2(18:1) | 1.20758 |
| TAG54:3(16:0) | 1.20582 |
| TAG51:3(15:0) | 1.20299 |
| PE34:2(16:0) | 1.20035 |
| LPE18:1 | 1.19728 |
| TAG48:4(16:1) | 1.19688 |
| LBPA36:4 | 1.19627 |
| TAG58:8(22:6) | 1.19114 |
| CE22:4 | 1.19102 |
| PC40:7(20:4) | 1.18876 |
| TAG54:8(22:6) | 1.18847 |
| PC40:7(20:3) | 1.1875 |
| TAG52:6(16:2) | 1.18617 |
| TAG54:2(18:0) | 1.18281 |
| TAG53:4(17:1) | 1.17747 |
| TAG56:6(20:4) | 1.17515 |
| PE36:4 | 1.17507 |
| PE36:1 | 1.17282 |
| TAG54:6(20:4) | 1.17064 |
| TAG56:2(20:1) | 1.1682 |
| TAG50:3(18:1) | 1.16572 |
| TAG56:8(22:5) | 1.16105 |
| PS40:4(20:4) | 1.16023 |
| TAG56:6(22:5) | 1.15419 |
| TAG46:1(16:1) | 1.15161 |
| TAG54:6(18:2) | 1.14569 |
| SM18:1/18:1 | 1.14405 |
| LPI16:1 | 1.14311 |
| TAG50:2(18:2) | 1.14062 |
| TAG58:8(20:4) | 1.13948 |
| LysoPC18:0 | 1.13702 |
| PS40:5(22:5) | 1.13323 |
| TAG58:7(20:4) | 1.13035 |
| TAG48:2(18:2) | 1.13019 |
| TAG54:4(16:0) | 1.12998 |
| TAG52:4(18:1) | 1.12848 |
| PG38:5 | 1.12839 |
| DAG36:2(18:2/18:0) | 1.12818 |
| PE40:6(20:3) | 1.12793 |
| TAG54:4(18:0) | 1.12514 |
| TAG52:2(18:2) | 1.12446 |
| TAG56:5(18:1) | 1.12275 |
| PE38:4p | 1.12255 |
| PE38:4(20:3) | 1.11973 |
| PC40:7(22:5) | 1.11834 |
| TAG54:5(16:0) | 1.11749 |
| PG36:4 | 1.11506 |
| LysoPC16:1 | 1.1146 |
| CE22:3 | 1.11338 |
| CE20:0 | 1.11201 |
| Cerd18:0/22:0 | 1.11137 |
| TAG56:5(22:4) | 1.11102 |
| PG36:3(18:1) | 1.10246 |
| SM18:1/20:0 | 1.10142 |
| CE17:1 | 1.10091 |
| TAG52:4(18:3) | 1.09754 |
| SM18:1/16:1 | 1.09382 |
| PI36:3(20:3) | 1.09359 |
| TAG53:4(17:0) | 1.0915 |
| TAG52:3(18:1) | 1.09129 |
| TAG52:3(18:2) | 1.08928 |
| PE40:4 | 1.08915 |
| SM18:1/24:1 | 1.08831 |
| PC34:1 | 1.08811 |
| LysoPC22:5 | 1.08287 |
| TAG53:3(17:0) | 1.08198 |
| PE34:2 | 1.08003 |
| TAG54:5(20:4) | 1.07886 |
| PC40:5p | 1.07831 |
| DAG32:2(18:2/14:0) | 1.07689 |
| PC38:3(20:3) | 1.07593 |
| TAG52:3(16:0) | 1.07571 |
| PC36:3(18:1) | 1.07503 |
| TAG58:7(22:5) | 1.07394 |
| TAG54:3(18:1) | 1.07382 |
| PC40:6p | 1.06852 |
| SM18:1/22:0 | 1.0681 |
| TAG56:5(18:2) | 1.06696 |
| TAG52:4(16:1) | 1.06607 |
| PC36:0p | 1.06473 |
| TAG54:5(16:1) | 1.06197 |
| TAG46:1(16:0) | 1.06111 |
| PE38:4 | 1.05992 |
| FA18:3 | 1.05834 |
| PE36:4(18:2) | 1.05618 |
| TAG50:4(18:2) | 1.05499 |
| LPS18:1 | 1.0537 |
| TAG52:4(16:2) | 1.05227 |
| TAG50:2(16:2) | 1.05053 |
| TAG54:7(18:2) | 1.04942 |
| PE38:5p(20:4) | 1.04719 |
| TAG52:5(16:0) | 1.04716 |
| PC38:4 | 1.04713 |
| TAG56:6(18:3) | 1.04207 |
| TAG48:2(18:1) | 1.04179 |
| TAG54:2(18:2) | 1.03699 |
| PI36:3 | 1.03661 |
| PE40:5 | 1.03392 |
| TAG48:1(18:0) | 1.03344 |
| TAG54:5(18:2) | 1.02752 |
| Cerd18:0/24:1 | 1.02716 |
| TAG56:6(20:3) | 1.0267 |
| LPS18:0 | 1.02221 |
| PE32:0 | 1.01829 |
| TAG54:3(18:2) | 1.01733 |
| TAG56:5(20:3) | 1.01731 |
| PS40:6(22:6) | 1.01726 |
| TAG56:4(18:1) | 1.01723 |
| CE22:1 | 1.01722 |
| PG36:1 | 1.01717 |
| TAG58:7(22:6) | 1.01555 |
| DAG36:3(18:2/18:1) | 1.01336 |
| TAG54:4(18:3) | 1.01265 |
| DAG40:6(18:1/22:5) | 1.01259 |
| TAG56:5(20:2) | 1.01052 |
| TAG56:3(22:1) | 1.00966 |
| GM3 18:0/18:0 | 1.00919 |
| CE18:1 | 1.00831 |
| TAG52:3(16:2) | 1.00752 |
| TAG56:2(18:1) | 1.00279 |
| TAG50:4(16:2) | 1.00053 |

Supplemental table 5. Concentration of lipid metabolites with FDR<0.05.

| Lipid metabolite | LVRR | No LVRR | P-value | FDR |
| --- | --- | --- | --- | --- |
| LysoPA16:0(µmol/L) | 1.58(1.24.3.33) | 0.68 (0.44,0.95) | 0.00011 | 0.014701 |
| LysoPA18:3(µmol/L) | 0.19(0.11,0.29) | 0.07(0.06,0.13) | 0.00023 | 0.036881 |
| LysoPA18:2(µmol/L) | 6.49(5.07,8.58) | 2.61(1.75,4.35) | 0.00009 | 0.014701 |
| LysoPA18:1(µmol/L) | 1.46(1.41,3.32) | 0.69(0.43,1.07) | 0.00010 | 0.014701 |
| LysoPA18:0(µmol/L) | 0.38(0.31,0.72) | 0.16(0.11,0.24) | 0.00009 | 0.014701 |

Variables were presented as median and interquartile range and compared using the Mann-Whitney U test.

LVRR, left ventricular remodeling reverse; FDR, false discovery rate.

Supplemental figure legend

Supplemental figure 1. Screen candidate metabolites of LVRR with OPLS-DA analysis (A) and random forest (B,C,D) in discovery set. (B) showed the number of decision trees; (C) Cross validation error curve of random forest; (D) displayed the importance of top 20 metabolites in order.

Supplemental figure 2.Spearman's correlation analysis ofage (month) and (A) LysoPA 16:0, (B) LysoPA 18:2, (C) LysoPA 18:1, (D) LysoPA 18:0. Raw levels of LysoPAs were lg-transformed and scaled (mean centered and divided by the standard deviation).
