# Supplementary figures and images for: Higher Serum Lysophosphatidic Acids Predict Left Ventricular Reverse Remodeling in Pediatric Dilated Cardiomyopathy
Source: Front Pediatr. 2021 Aug 16;9:710720. doi: 10.3389/fped.2021.710720 (PMC8415784; doi:10.3389/fped.2021.710720)

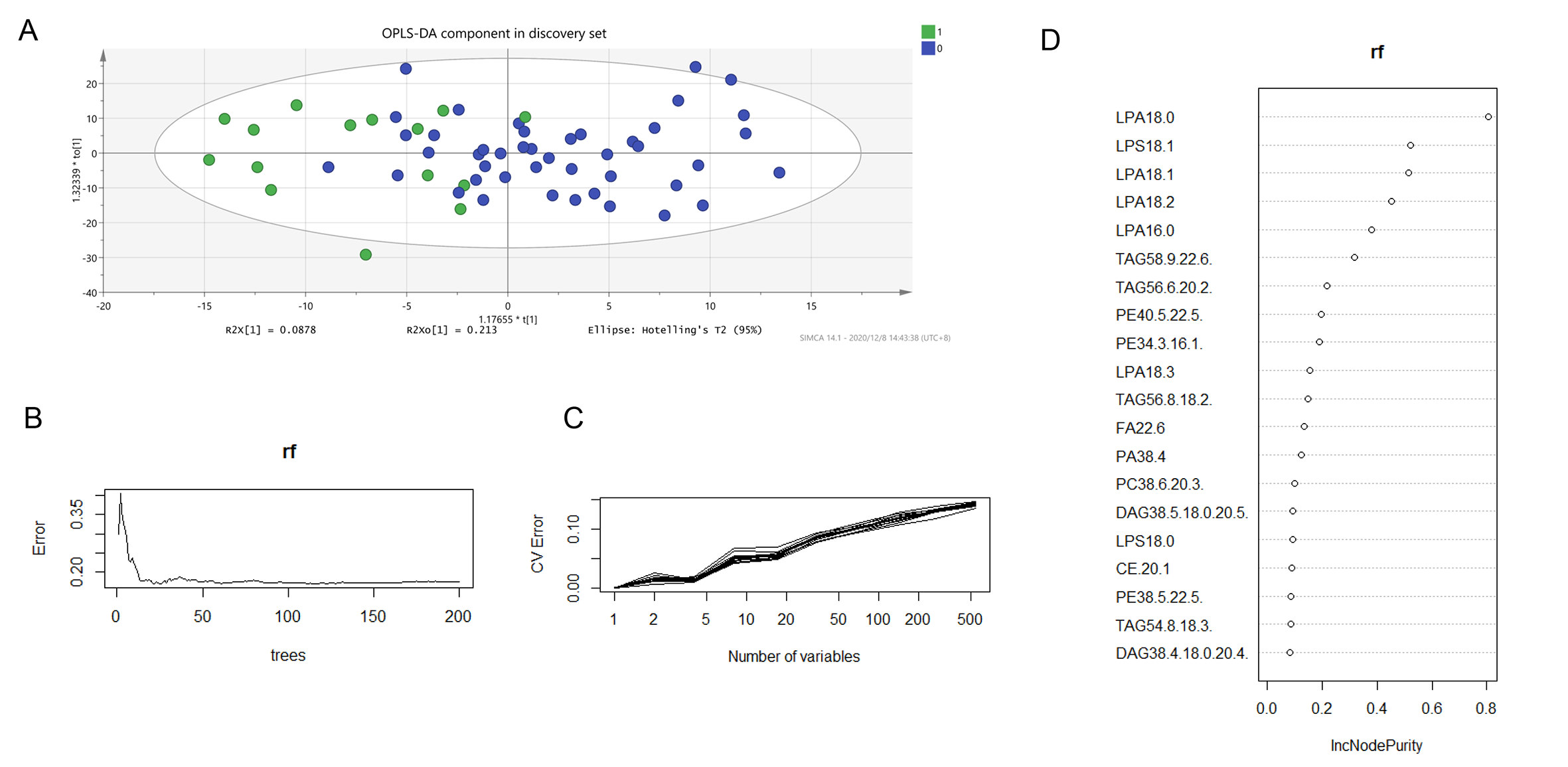

Supplement: Supplementary file 2 [file Image_1.TIF]

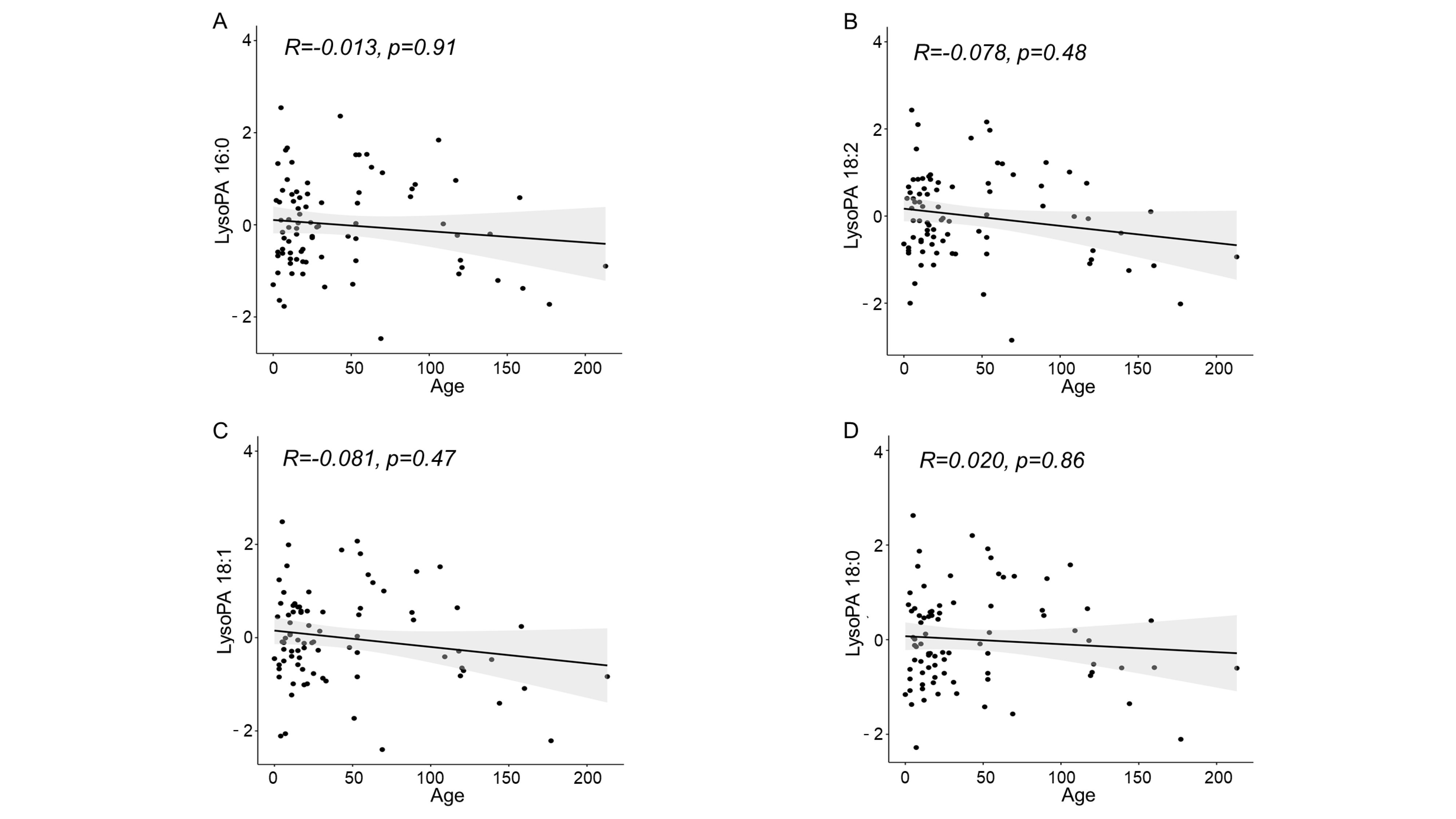

Supplement: Supplementary file 3 [file Image_2.TIF]
